# Interactions between PCSK9 and NLRP3 inflammasome signaling in atherosclerosis

**DOI:** 10.3389/fimmu.2023.1126823

**Published:** 2023-02-22

**Authors:** Yanan Wang, Dan Fang, Qinzhi Yang, Jingcan You, Liqun Wang, Jianbo Wu, Min Zeng, Mao Luo

**Affiliations:** ^1^ Key Laboratory of Medical Electrophysiology, Ministry of Education, Drug Discovery Research Center, Southwest Medical University, Luzhou, Sichuan, China; ^2^ Laboratory for Cardiovascular Pharmacology, Department of Pharmacology, School of Pharmacy, Southwest Medical University, Luzhou, Sichuan, China; ^3^ Metabolic Vascular Disease Key Laboratory of Sichuan Province, Luzhou Municipal Key Laboratory of Thrombosis and Vascular Biology, Luzhou, Sichuan, China; ^4^ Department of Pharmacy, The Affiliated Hospital of Southwest Medical University, Luzhou, Sichuan, China

**Keywords:** PCSK9, NLRP3 inflammasome signaling, interactions, atherosclerosis, therapy

## Abstract

Atherosclerosis is an early pathological basis of numerous cardiovascular events that result in death or disability. Recent studies have described PCSK9 as a novel target for the treatment of atherosclerosis; PCSK9 is capable of degrading LDLR on the surface of hepatocytes through the regulation of lipid metabolism, and it can function as a novel inflammatory modulator in atherosclerosis. Inflammasomes are important intracellular multiprotein complexes that promote the inflammatory response in atherosclerosis. Among inflammasomes, the NLRP3 inflammasome is particularly notable because of its important role in the development of atherosclerotic disease. After activation, NLRP3 forms a complex with ASC and pro-caspase-1, converting pro-caspase-1 into activated caspase-1, which may trigger the release of IL-1β and IL-18 and contribute to the inflammatory response. Several recent studies have indicated that there may be interactions between PCSK9 and the NLRP3 inflammasome, which may contribute to the inflammatory response that drives atherosclerosis development and progression. On the one hand, the NLRP3 inflammasome plays an important role *via* IL-1β in regulating PCSK9 secretion. On the other hand, PCSK9 regulates caspase-1-dependent pyroptosis by initiating mtDNA damage and activating NLRP3 inflammasome signaling. This paper reviews the mechanisms underlying PCSK9 and NLRP3 inflammasome activation in the context of atherosclerosis. Furthermore, we describe the current understanding of the specific molecular mechanism underlying the interactions between PCSK9 and NLRP3 inflammasome signaling as well as the drug repositioning events that influence vascular cells and exert beneficial antiatherosclerotic effects. This review may provide a new therapeutic direction for the effective prevention and treatment of atherosclerosis in the clinic.

## Introduction

Inflammation is an important driver of atherosclerosis, which is an early pathological basis of cardiovascular disease (1). Atherosclerosis is characterized by the excessive accumulation of lipids, extracellular matrix, and cholesterol-laden macrophages under the arterial endothelium, resulting in the formation of atherosclerotic plaques (2, 3). LDL-cholesterol (C) is considered to be a major risk factor for the development of atherosclerotic diseases (4). The mechanism by which proprotein convertase subtilisin/kexin type 9 (PCSK9) regulates LDLR degradation involves both extracellular and intracellular pathways. On the one hand, extracellular PCSK9 can act as a companion protein and bind to LDL-receptor (R) on the cell surface, thus causing the formation of a complex and directing LDLR to lysosomes where the PCSK9/LDLR complex is degraded, thereby promoting further LDL-C accumulation (5). On the other hand, intracellular PCSK9 can also directly bind with LDLR in the Golgi network to induce the lysosomal degradation of LDLR (6). PCSK9 is closely associated with the indirect regulation of lipid metabolism (5) and participates in the direct regulation of atherosclerosis *via* the accumulation of foam cells and inflammatory mediators as well as apoptosis in vascular walls (7). If PCSK9 inhibition is reduced, there is more PCSK9, more degraded LDLRs and therefore an increase in LDL levels (8). Persistent accumulation of LDL results in not only the formation of foam cells but also the chronic amplification of inflammatory responses, which are major causes of plaque rupture and vascular thrombosis (9). NOD-like receptor thermal protein domain associated protein 3 (NLRP3) inflammasomes are classical receptors of intracellular innate immunity that closely regulate inflammatory responses (10). NLRP3 inflammasome activation is a powerful mediator of the inflammatory response *via* caspase-1 activation (11). The NLRP3 inflammasome can activate caspase-1 and cleave pro-IL-1β and IL-18 to generate IL-1β and IL-18, which further promote inflammatory responses and play crucial roles in regulating atherosclerotic lesions (12).

Recently, several studies have reported that PCSK9 activates the NLRP3 inflammasome signaling pathway and the associated inflammation (13–15). Conversely, the NLRP3 inflammasome signaling pathway can regulate PCSK9 secretion (16). However, the regulatory mechanisms are still not fully understood, especially in the context of atherosclerosis. In this review, we summarize the current findings related to the interactions between PCSK9 and the NLRP3 inflammasome in atherosclerosis. Furthermore, we describe the specific molecular mechanism underlying the PCSK9 and NLRP3 inflammasome signaling pathway in atherosclerosis and cells related to inflammation, including vascular smooth muscle cells (VSMCs), endothelial cells (ECs) and macrophages (MФ). These findings may provide a novel theoretical basis and important targets for clinical application in the treatment of atherosclerosis.

## PCSK9 biology and its role in atherothrombosis

### PCSK9 biology

**Figure 1:**
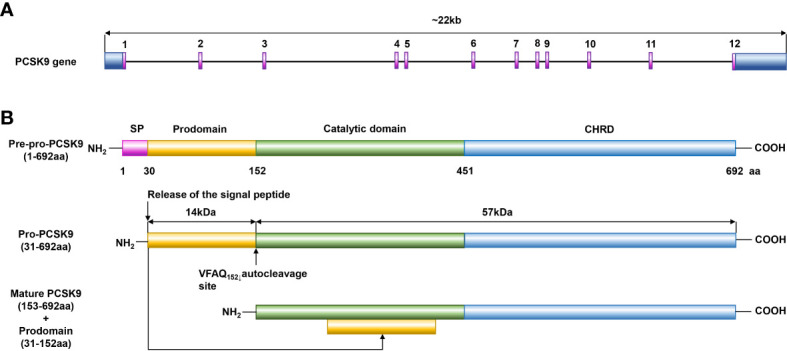
Structure diagram of PCSK9. **(A)** PCSK9 is the ninth member of the subtilisin serine protease family, and its gene is located on human chromosome 1p32.3, is 22 kb in length, includes 12 exons, and encodes 692 amino acids. **(B)** PCSK9 consists of a signal peptide (residues 1–30), a prodomain (residues 31–152), a catalytic domain (residues 153–451), and a C-terminal domain (residues 452–692). Similar to other proprotein convertases, PCSK9 is synthesized as a soluble proenzyme and undergoes autocatalytic cleavage in the ER at residue 152 between its prodomain and catalytic domains. Then, a stable heterodimer consisting of a prodomain of approximately 14 kDa and a mature fragment of approximately 57 kDa is formed, and after transportation to the Golgi apparatus, the protein is modified by acetylation and subsequently secreted.

In 2003, Abifadel M et al. first reported that PCSK9 is highly expressed in the liver and contributes to cholesterol homeostasis (17). Similar to other proprotein convertases, PCSK9 is synthesized as a soluble proenzyme and undergoes autocatalytic cleavage in the endoplasmic reticulum (ER) at residue 152 between its prodomain and catalytic domains (18). Then, a stable heterodimer consisting of a prodomain of approximately 14 kDa and a mature fragment of approximately 57 kDa is formed, as shown in **Figure 1**. After transportation to the Golgi apparatus, the protein is modified by acetylation and subsequently secreted (18). PCSK9 is mainly secreted by the liver, small intestine, kidney, skin, and cerebrospinal fluid, while PCSK9 in blood is almost exclusively secreted from the liver (19). PCSK9 and LDLR form a tight complex and are targeted to lysosomes for degradation, thereby reducing the level of LDLR on the surface of hepatocytes and decreasing hepatic clearance of LDL-C (20).

Through its function of degrading the LDLR, PCSK9 is a key player in lipid metabolism by regulating the LDL level in blood responsible for hypercholesterolemia, which is associated with the risk of atherosclerosis (21). Overexpression of PCSK9 was found to inversely downregulate the expression of LDLR and reduce the clearance of cholesterol from plasma, so inhibiting PCSK9 overexpression is significant in the prevention and treatment of atherosclerosis. Moreover, Tavori et al. found that PCSK9 expression is involved in regulating the size and composition of atherosclerotic plaques and significantly enlarges atherosclerotic lesion areas (8). Giunzioni et al. further showed that PCSK9 directly increases the inflammation of atherosclerotic lesion in an LDLR-dependent but cholesterol-independent manner, suggesting that therapeutic PCSK9 inhibition may have vascular benefits that are secondary to reductions in the LDL levels (22). Overall, PCSK9 is expressed at high levels at the sites of atherosclerotic lesions, particularly in intimal plaques (23). PCSK9 inhibition has emerged as a potential novel therapeutic approach to treat hypercholesterolemia and associated diseases, such as atherosclerosis.

## PCSK9 is a key modulator of atherosclerosis

**Figure 2:**
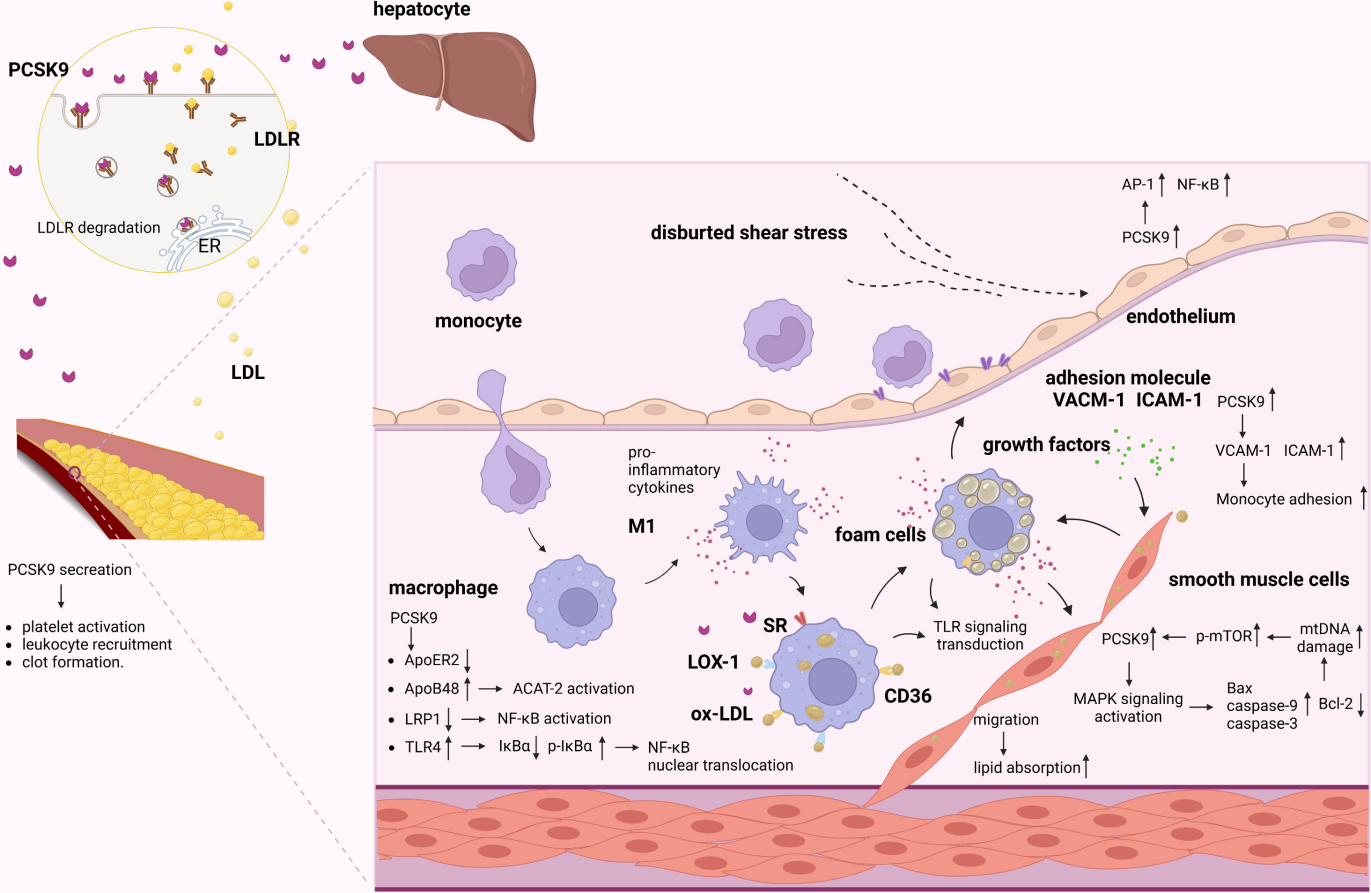
Role of PCSK9 in atherosclerosis. PCSK9 and LDLR form a complex and are targeted to lysosomes for degradation. Secreted PCSK9 by the liver reduces the level of LDLR not only on hepatocytes surface but also on other cells. PCSK9 promotes platelet activation, leukocyte recruitment and clot formation both in the plasma and in vascular cells. Proinflammatory stimuli activate ECs and increase adhesion molecules, promoting the adhesion and migration of monocytes. Migrated monocytes and ox-LDL-C accumulate in the subintimal region. Meanwhile, migrated monocytes transforms into MФs. In inflammatory milieu, PCSK9 increases scavenger receptors to increase ox-LDL uptake. In addition, PCSK9 mediated inflammation *via* activating LRP1, TLR4/NF-κB pathway. PCSK9 increased cholesterol synthesis by activating ACAT-2/ApoB48. Furthermore, migrated SMCs can absorb lipid and exacerbate the inflammatory responses and myogenic foam cell formation caused by the effects of growth factors and proinflammatory cytokines. In VSMCs, PCSK9 is enhanced by mtDNA damage *via* the upregulation of p-mTOR. In turn, PCSK9 increases mtDNA damage by regulating the apoptotic proteins *via* MAPK signaling pathway. Besides, low shear stress increases PCSK9, which induces EC dysfunction and upregulates AP-1 and NF-κB.

It has been clearly established that PCSK9 plays a crucial role in the development of atherosclerosis. While the liver is the major source of circulating PCSK9, PCSK9 is also expressed in various cellular components of atherosclerotic plaques, including monocytes/MФs, VSMCs, and ECs, that directly participate in the progression of atherosclerotic lesions by exacerbating vascular inflammation (**Figure 2** and **Table S1**) (24–31). Moreover, the secretion of PCSK9 in ECs, SMCs and MФs were strongly induced by LPS treatment with or without ATP compared with the control (23, 32). PCSK9 secreted by VSMCs, which express more PCSK9 than ECs in a paracrine manner, downregulating LDLR expression on the cell surface of MФs and preventing the formation of foam cells, thus reducing atherosclerosis progression. However, native LDL molecules are not the major source of cholesterol accumulation in macrophages and LDLR is not the main receptor for lipoprotein uptake in cells within the atherosclerotic plaque (5). Scavenger receptors expressed by MФ in vessel walls take up LDL-cholesterol, resulting in the transformation of MФs into foam cells that secrete a large amount of proinflammatory cytokines and mature into activated MФ, contributing to the acceleration of arterial inflammation and atherosclerosis (33, 34). Cluster of differentiation 36(CD36) is a scavenger receptor that is highly expressed in macrophages and macrophage-derived foam cells in atherosclerotic plaques. Levy et al. found that gain-of-function PCSK9 mutants could significantly increase CD36 expression (35). In addition, Ding et al. also showed that PCSK9 can enhance the uptake of ox-LDL in MФs by targeting lectin-like ox-LDL receptor 1(LOX-1) (23). Moreover, Giunzioni et al. found that PCSK9 exerts a direct effect on the monocyte/MФ phenotype during the progression of atherosclerosis (22). Given that LDL-R-related protein 1(LRP1) deficiency induces nuclear factor kappa-B (NF-κB) activation and promotes a proinflammatory phenotype in MФs, some studies have suggested that PCSK9 induces macrophage inflammation by targeting LRP1 for degradation. Tang et al. found that PCSK9 overexpression upregulates toll-like receptors 4 (TLR4) expression and promotes inhibitor kappa B alpha (IκBα) degradation, p-IκBα expression, and NF-κB nuclear translocation (36). The findings show that PCSK9 enhances inflammatory cytokine secretion through TLR4/NF-κB pathway activation in MФs. Another mechanism underlying the PCSK9-mediated promotion of inflammation might involve the PCSK9-induced LDLR-independent degradation of apolipoprotein E receptor 2 (ApoER2) (37), which plays a critical role in maintaining the anti-inflammatory phenotype of MФs; these results indicate that PCSK9 can regulate macrophage inflammation through inflammasome activation.

VSMCs are important for the maintenance of vascular homeostasis and play a key role in atherosclerosis (38). PCSK9 is reported to be expressed in VSMCs in atherosclerotic plaques (39), and its expression is markedly increased by inflammatory stimuli, indicating that proinflammatory factors that are associated with atherosclerosis stimulate PCSK9 expression in VSMCs and suggesting a critical role for PCSK9 in the development of atherosclerotic lesions (40). For example, migration of SMCs from the tunica media of the vessel wall can lead to increased lipid absorption and exacerbate the inflammatory responses and myogenic foam cell formation caused by the effects of growth factors and proinflammatory cytokines on the fatty streak (41). Previous studies have shown that there is a positive feedback interplay between VSMC-derived PCSK9 and mitochondrial DNA (mtDNA) damage in the proinflammatory milieu that involves mitochondrial reactive oxygen species (mtROS); this feedback loop results in inflammation, oxidative stress, and apoptosis, which directly contribute to atherosclerosis (42). In VSMCs, PCSK9, which increases mtDNA damage by upregulating the expression of the pro-apoptotic proteins Bax, caspase-9, and caspase-3 and downregulating the expression of Bcl-2, is correlated with the activation of the MAPK signaling pathway. Moreover, PCSK9 expression is enhanced by mtDNA damage *via* the upregulation of p-mTOR expression in a dose-dependent manner, while inhibition of PCSK9 reduces mammalian target of rapamycin (mTOR) phosphorylation and decreases VSMC autophagy.

Vascular endothelial dysfunction and the inflammatory response contribute to the initiation and progression of atherosclerosis (43). Landlinger et al. found that treatment of mice with AT04A obviously suppressed the inflammatory response and activated ECs and stimulated monocyte/macrophage migration, suggesting that PCSK9 inhibition also suppresses endothelial inflammation (44). Although PCSK9 expression and secretion by ECs may be lower than that by VSMCs, the effects of PCSK9 on EC biology in atherosclerosis cannot be ignored. The development of atherosclerosis is caused by a variety of factors, including biochemical factors and physical factors. Hemodynamic shear stress regulates EC functions and influences the pathobiology of atherosclerosis (45). Laminar shear stress is thought to exert atheroprotective effects (46). Conversely, low shear stress significantly increases PCSK9 expression, induces EC dysfunction, and upregulates the expression of transcription factors, including AP-1 and NF-κB, that promote pro-oxidant and proinflammatory states (40, 46); these results indicate that PCSK9 may play an important role in the EC inflammation that is induced by low shear stress.

## NLRP3 inflammasome signaling pathway

Hosts regulate the release of intracellular inflammatory mediators and the initiation of inflammatory responses mainly through two types of pattern recognition receptors (PRRs), namely, membrane-bound Toll-like receptors (TLRs) and nucleotide-binding oligomerization domain (NOD)-like receptors (NLRs) (47, 48); these receptors recognize pathogen-associated molecular patterns (PAMPs) and danger-associated molecular patterns (DAMPs) and thus promote the maturation and release of tightly regulated, highly inflammatory cytokines. NLRP3 is a recognized PRR, and it is a cytoplasmic receptor that responds to danger signals and can be activated to form the NLRP3 inflammasome (49).

**Figure 3:**
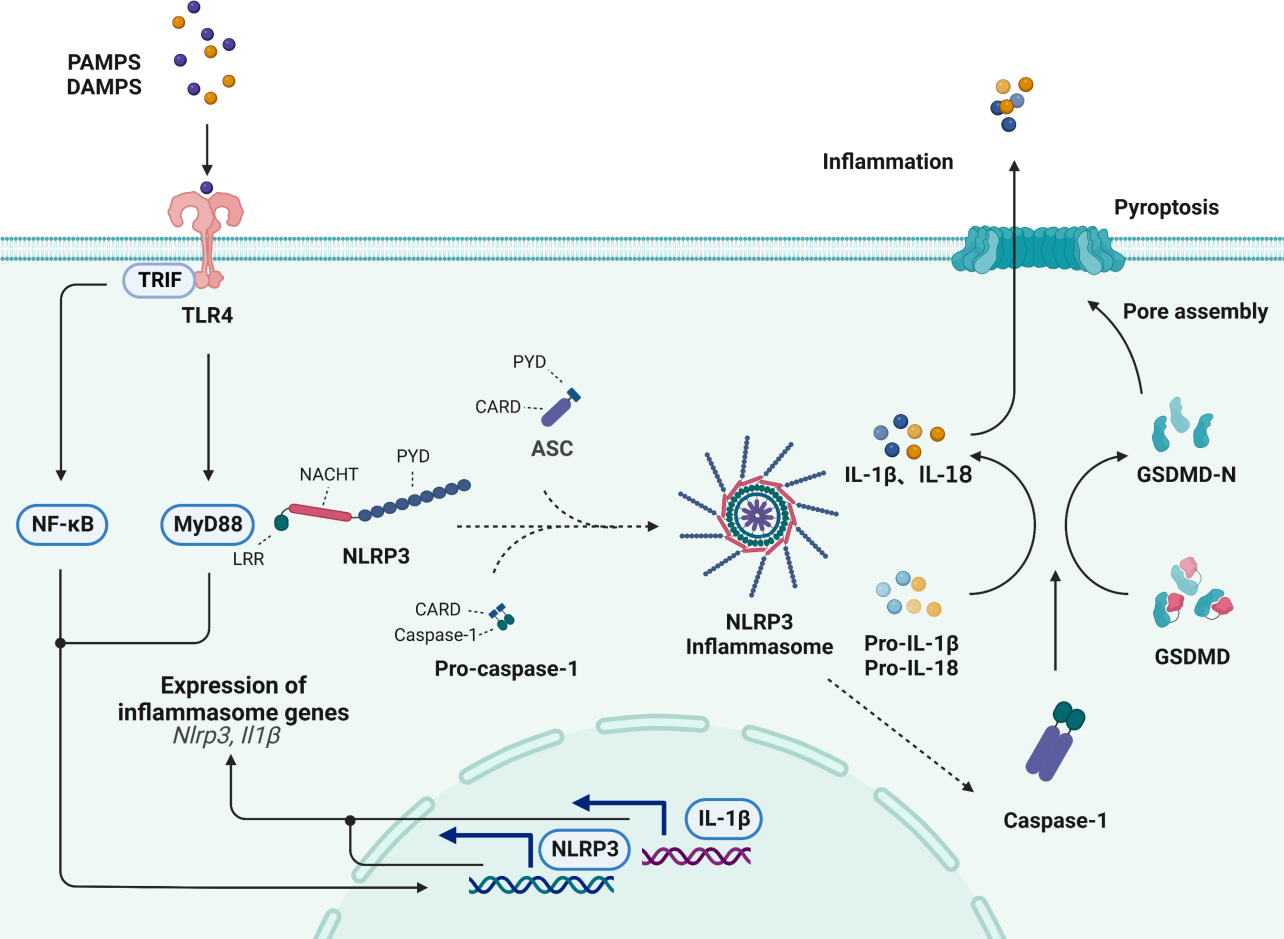
Activation of the NLRP3 inflammasome signaling pathway. The NLRP3 inflammasome is comprised of the NLRP3, ASC and pro-caspase-1. The sensing of various stimuli by TLRs on the cell membrane can induce the activation of the TRIF/NF-κB, the activation of TLR4/MyD88 signaling, the transcription of NLRP3 receptor proteins and lead to formation of NLRP3 inflammasome. Upon activation, NLRP3 induces the proteolytic activation of caspase-1, which facilitates the cleavage of pro-IL-1β and pro-IL-18. Additionally, activated caspase-1 cleaves GSDMD and generates an active GSDMD-NT, which translocates to the plasma membrane and oligomerizes to form a pore that allows the secretion of IL-1β and IL-18, subsequently inducing pyroptotic cell death.

The NLRP3 inflammasome is a multimolecular protein complex that comprises the NOD-like receptor NLRP3, the adaptor ASC and the effector pro-caspase-1, which play fundamental roles in inflammation (50). Among these components, the NOD-like receptor NLRP3 contains a leucine-rich repeat (LRR) domain, nucleotide triphosphatase (NACHT) domain, and pyrin domain (PYD), and it plays fundamental roles in inflammation (51). ASC is composed of the N-terminal PYD domain and the C-terminal CARD domain, which promote oligomeric homotypic interactions (52). Pro-caspase-1 is composed of a CARD and catalytic domain, including p10 and p20 (53). The sensing of various stimuli by TLRs on the cell membrane can induce the activation of the TRIF/NF-κB and TLR4/MyD88 signaling pathways and upregulate the transcription of NLRP3 receptor proteins and proinflammatory cytokines (54, 55). Moreover, the LRR domain of NLRP3 senses stimuli, which leads to NACHT domain oligomerization, most likely by promoting a PYD-ASC interaction, and these processes result in the formation of the NLRP3 inflammasome *via* the binding of pro-caspase-1, which is another component of the NLRP3 inflammasome, to CARD and ASC (56, 57). Upon activation, the NLRP3 inflammasome results in the proteolytic activation of caspase-1, which facilitates the cleavage of pro-IL-1β and pro-IL-18 and the secretion of the proinflammatory cytokines IL-1β and IL-18 (58) (**Figure 3**).

Additionally, the caspase-1-dependent cleavage of gasdermin-D (GSDMD) is thought to be the classic initiator pyroptotic cell death (59). Pyroptosis is a novel form of cell death that is induced in response to cell disruption by the inflammasome-induced GSDMD protein, which promotes the release of proinflammatory cytokines, such as IL-1β and IL-18 (60). Previous studies have revealed that pyroptosis is involved in atherosclerotic plaque formation. Activated caspase-1 cleaves GSDMD and generates an active N-terminal cleaving product (GSDMD-NT), which translocates to the plasma membrane and oligomerizes to form a pore that allows the release of IL-1β and IL-18, subsequently inducing pyroptotic cell death and promoting the occurrence and development of atherosclerosis (61).

## The role of the NLRP3 inflammasome signaling pathway in atherosclerosis

**Figure 4:**
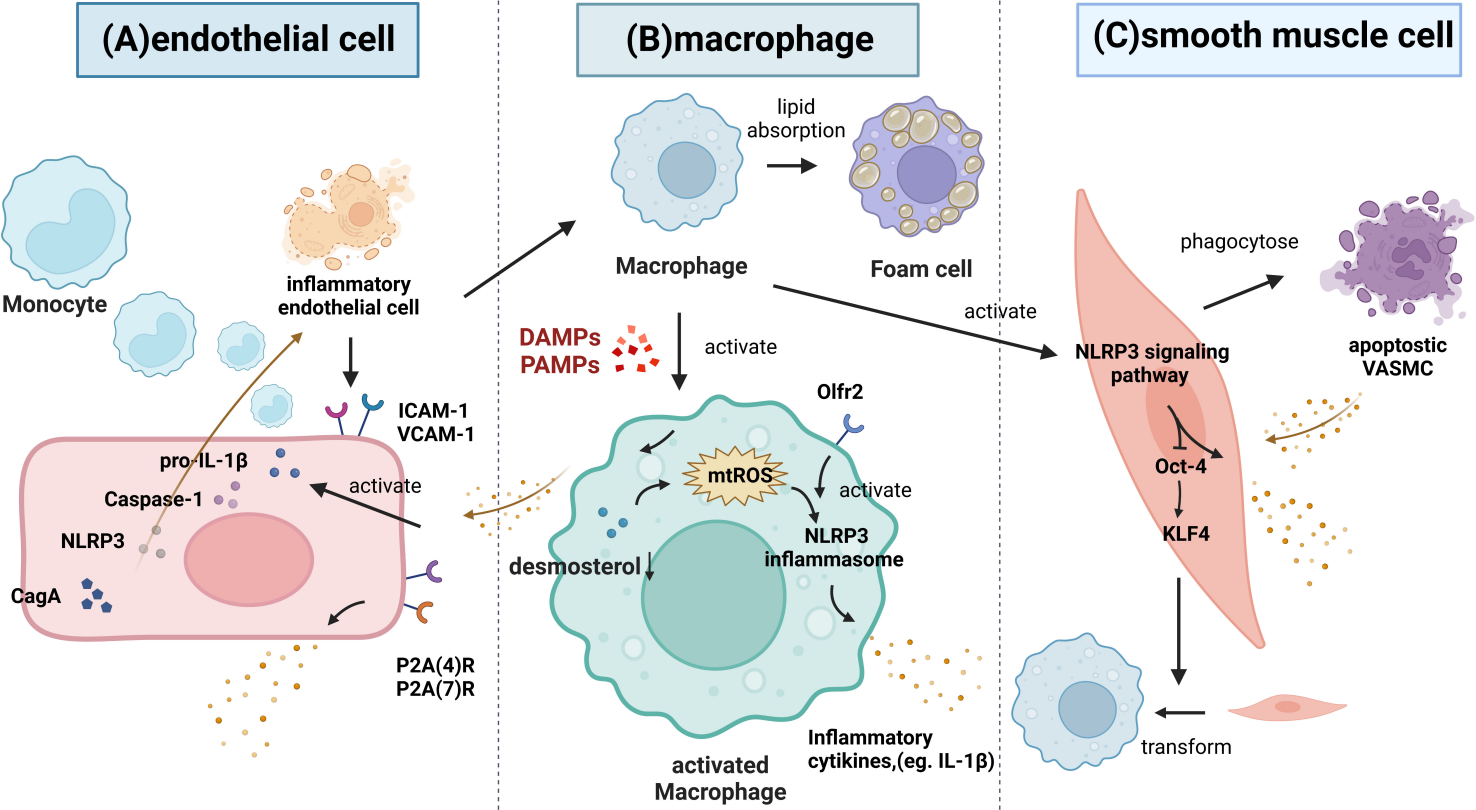
The role of the NLRP3 inflammasome signaling pathway in atherosclerosis. **(A)** Inflammatory endothelium can improve the recruitment of leucocytes *via* increasing ICAM-1 and VCAM-1, thus attracting monocytes to migrate and differentiate into macrophages. Then, macrophages phagocytose the modified lipoproteins and increase the foam cells formation. Inflammatory stimuli activate the synthesis of pro-IL-1β and increase the caspase-1. Both of P2X(4)R and P2X(7)R are also significantly upregulated under inflammatory conditions and promote the release of IL-1β and IL-1Ra. Moreover, CagA promotes endothelial inflammation through the NLRP3/caspase-1/IL-1β axis. **(B)** Various PAMPs and DAMPs can activate macrophages. Macrophages express the Olfr2, which drive atherosclerosis *via* NLRP3-dependent IL-1β secretion. Desmosterol accumulation in mitochondria suppresses mtROS production and NLRP3 inflammasome activation. **(C)** VSMC apoptosis can induce the inflammatory response. Normal VSMCs inhibit the inflammation by phagocytosing apoptotic VSMCs *in vivo*. Monocytes can trigger NLRP3 inflammasome activation and the expression of NLRP3, promoting IL-1β secretion, and these cells can decrease Oct-4 in VSMCs and upregulate klf4 to control the phenotypic transformation of VSMCs into macrophage-like cells.

Recently, it has been shown that NLRP3 inflammasome/IL-1β signaling-mediated inflammation plays an important role in the development of atherosclerosis (12), as is shown in **Table S2** (62–73) and **Figure 4**. The inflammatory process starts with inflammasome activation, which leads to the release of mature IL-1β. Various PAMPs and DAMPs have been shown to activate macrophages (74). Upon activation, macrophages exacerbate the vascular inflammatory response by releasing cytokines, and activated macrophages are the main source of IL-1β (75). For example, Orecchioni et al. recently found that macrophages express the olfactory receptor Olfr2 and all associated trafficking and signaling molecules, which drive atherosclerosis *via* NLRP3-dependent IL-1β secretion (76). In addition, a study by Zhang et al. reported that desmosterol suppressed inflammasome activation in macrophages and protected against vascular inflammation and atherosclerosis (77). Decreased desmosterol accumulation in mitochondria promotes mtROS production and NLRP3-dependent inflammasome activation. NLRP3 or ASC deficiency can reverse the increase in inflammasome activity and atherogenesis that is observed in desmosterol-depleted macrophages.

Endothelium dysfunction is involved in the development of atherosclerotic vascular lesions. It has been shown that ECs synthesize IL-1β in response to inflammatory stimuli; moreover, the atherosclerotic endothelium exhibits increased expression of IL-1β (78). Wilson et al. found that HUVECs express P2X (4) R and P2X(7)R subtypes, and both were significantly upregulated under inflammatory conditions (79). Activation of P2X(7)Rs results in the release of low levels of bioactive IL-1β and the simultaneous release of IL-1Ra (79). Wu et al. demonstrated that cytotoxin-associated gene A (CagA) promotes aortic endothelial inflammation and accelerates atherosclerosis through the NLRP3/caspase-1/IL-1β axis (80). In addition, the activation of the vascular endothelium and the infiltration of circulating monocytes into the vessel wall are considered to be key factors in the occurrence and development of atherosclerosis (81). Hettwer et al. revealed that IL-1β suppression reduces inflammatory leukocyte production and uptake in atherosclerosis (82). When the NLRP3 inflammasome is inhibited in ECs from atherosclerotic aortas, these cells show decreased expression of leukocyte chemoattractants and adhesion molecules, indicating that NLRP3 inflammasome- and IL-1β-targeted therapies may reduce blood leukocyte recruitment to atherosclerotic aortas.

The inflammatory response that is induced by VSMC apoptosis accounts for approximately 15% of the systemic inflammatory response in atherosclerosis. Apoptotic VSMCs are phagocytosed by normal endothelial cells *in vivo*, which does not require the involvement of macrophages, leading to the inhibition of inflammation and slowing the progression of atherosclerosis (83). In addition, Clarke et al. found that necrotic VSMCs release IL-1a, whereas apoptotic VSMCs undergoing secondary necrosis release both IL-1a and IL-1β. IL-1 from necrotic VSMCs induces the surrounding viable VSMCs to produce proinflammatory cytokines. Thus, the failure to clear apoptotic VSMCs caused by hyperlipidemia *in vivo* may promote the increases in serum cytokine levels and chronic inflammation associated with atherosclerosis (83). In addition, NLRP3 inflammasome activation and IL-1β signaling play a direct role in VSMC phenotypic switching (84). Fabienne Burger et al. found that monocytes can trigger NLRP3 transcription factors expression, thus promoting NLRP3 inflammasome activation and IL-1β secretion, and these cells can decrease Oct-4 expression in VSMCs and upregulate klf4 expression to control the phenotypic transformation of atherosclerotic VSMCs into macrophage-like cells (85).

## Interactions between PCSK9 and the NLRP3 inflammasome signaling pathway

### Effects of PCSK9 on the NLRP3 inflammasome signaling pathway

**Figure 5:**
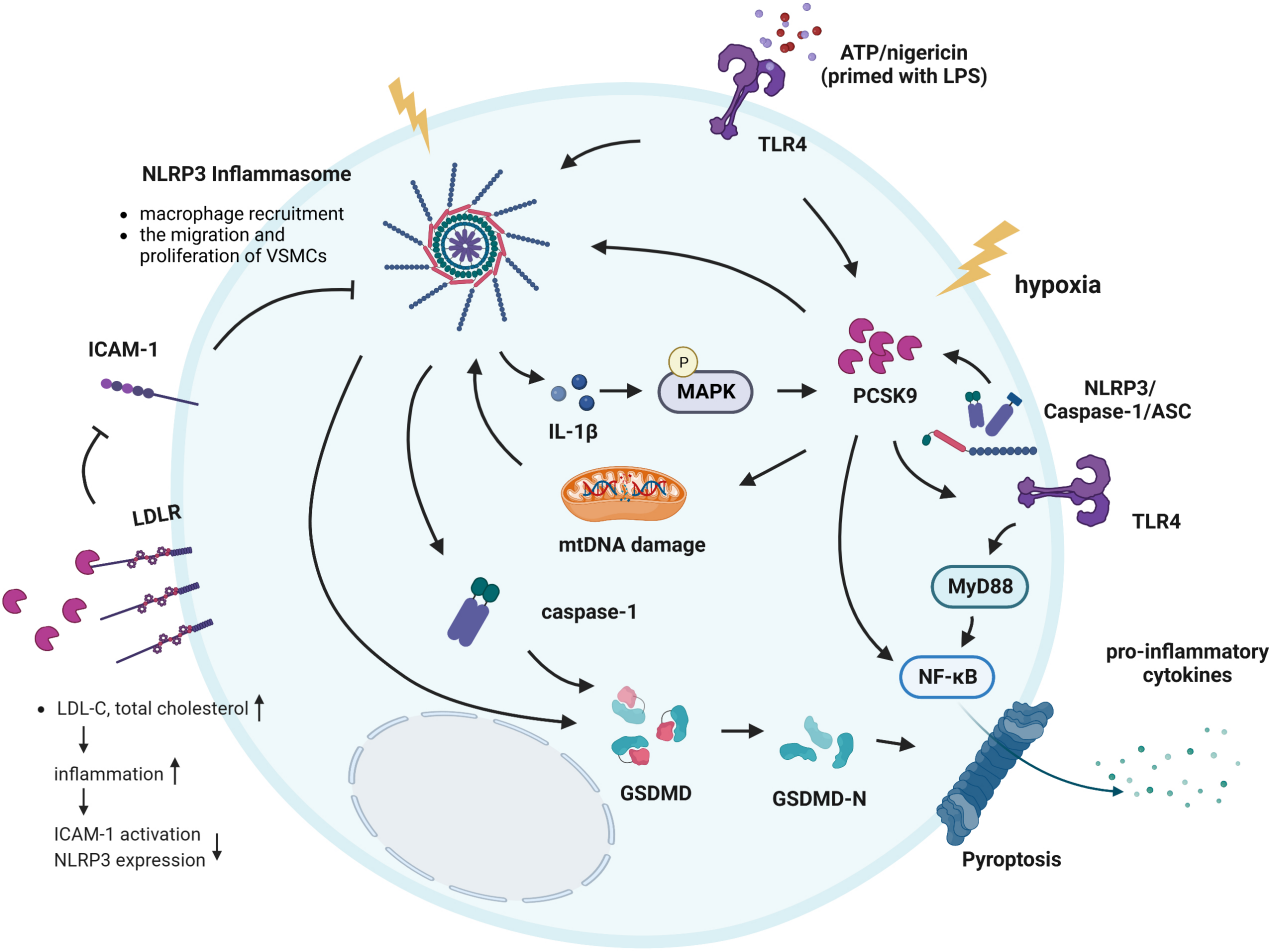
Interactions between PCSK9 and the NLRP3 inflammasome signaling pathway. ATP and nigericin simultaneously induced the NLRP3 inflammasome in cells primed with LPS and induced PCSK9 expression. In addition, hypoxia induces PCSK9 expression and NLRP3 inflammasome activation. The NLRP3 inflammasome and its downstream signals all regulate PCSK9 secretion, while MAPKs play a key role in regulating IL-1β-mediated PCSK9 secretion. PCSK9 can directly activate the NLRP3 inflammasome and promote the secretion of pro-inflammatory cytokines *via* NF-κB signaling. PCSK9 knockout inhibits macrophage recruitment, suppresses the migration and proliferation of VSMCs; these effects are related to NLRP3 inflammasome. PCSK9 initiates mtDNA damage, activates NLRP3 inflammasome signaling and subsequently induces pyroptosis. Another mechanism underlying the effects of PCSK9 on NLRP3 inflammasome activation involves TLR4/MyD88/NF-κB signaling. PCSK9 binds to LDLR, causing an increase in the total cholesterol and LDL-C levels, inducing inflammation, resulting in ICAM expression and NLRP3 inflammasome activation.

Several studies have revealed that both PCSK9 expression and NLRP3 inflammasome-induced pyroptosis occur in atherosclerotic plaques (13). Some studies have reported that there are interactions between PCSK9 and the NLRP3 inflammasome in atherosclerosis (86) (**Table S3**) (87, 88, **Figure 5**). PCSK9 is considered to be a key inflammatory signal, and it is also abundantly expressed in cells that are exposed to inflammatory stimuli, such as lipopolysaccharide (LPS) (89). The NLRP3 inflammasome has been considered to be a link between lipid metabolism and inflammation, and it induces pyroptosis and exerts a significant effect on atherogenesis (90). Under hypoxic conditions, PCSK9 promotes the secretion of proinflammatory cytokines by macrophages to exacerbate hypoxia/reoxygenation-induced cardiomyocyte injury by activating the NF-κB signaling pathway (91). Moreover, PCSK9 knockout significantly inhibits hypoxia-induced inflammation and cell death, inhibits macrophage recruitment, and suppresses the migration and proliferation of VSMCs; these effects are related to the inhibition of NLRP3 inflammasome activation (15). Using sepsis models in HUVECs and mice, Huang et al. found that increased PCSK9 expression during sepsis activates the TLR4/MyD88/NF-κB and NLRP3 pathways to induce inflammation, which results in vascular endothelial dysfunction and decreased survival (14). In addition, several studies have also demonstrated that the NLRP3 inflammasome and pyroptosis-related proteins are activated under hypoxia conditions (92). Ding et al. established a LCA ligation model in mice to model the atherosclerotic state and found that PCSK9 expression could be strongly enhanced in the zone bordering the infarcted area (93). Moreover, the study reported that pyroptosis is significantly observed in the zone bordering the myocardial infarcted area (94). These studies further demonstrated a close correlation between PCSK9 and the NLRP3 inflammasome signaling pathway, and both are involved in the process of hypoxia-induced pyroptosis.

Mitochondrial ROS promote macrophage pyroptosis by inducing GSDMD oxidation. Excess production of mtROS and subsequent mtDNA damage are common triggers of NLRP3 inflammasome activation (95), while GSDMD activation and LDH release are key mediators of the occurrence of pyroptosis. Wang et al. found that PCSK9 regulates pyroptosis *via* mtDNA damage in chronic myocardial ischemia (13). PCSK9 initiates mitochondrial DNA (mtDNA) damage, activates NLRP3 inflammasome signaling (NLRP3, ASC, Caspase-1, IL-1β, and IL-18), and subsequently induces Caspase-1-dependent pyroptosis, with intense expression of PCSK9 and the pyroptosis marker GSDMD-NT in the zone bordering the infarct area. Moreover, PCSK9 knockout significantly decreases the NLRP3 inflammasome signaling, GSDMD-NT expression, and LDH release (13, 15). These results suggest that PCSK9 regulates Caspase-1-dependent pyroptosis *via* mtDNA damage and may reveal proinflammatory processes, including NLRP3 inflammasome signaling and pyroptosis, as potential targets for the treatment of PCSK9-related cardiovascular diseases (13). Additionally, PCSK9 binds to the LDLR and enhances its degradation, which leads to the reduced clearance of LDL-C and a higher risk of atherosclerosis. Landlinger et al. found that AT04A immunization induced high and persistent levels of antibodies against PCSK9, significantly reducing the total cholesterol and LDL-C levels in plasma, decreasing vascular inflammation, and ultimately reducing ICAM expression in activated endothelial cells and caspase-1-activating NLRP3 inflammasome-related protein expression in proinflammatory macrophages (44).

### Effects of the NLRP3 inflammasome signaling pathway on PCSK9

Notably, the expression of NLRP3 inflammasome-related proteins and secretion of PCSK9 were particularly evident when mouse peritoneal macrophages were exposed to LPS, ATP or nigericin, indicating that the NLRP3 inflammasome is connected to PCSK9 secretion (16, 96). Macrophage-derived PCSK9 may also play an important role in atherogenesis *via* its local effects on atherosclerotic plaques (36). Ding et al. revealed that NLRP3 and its downstream signaling proteins ASC, Caspase-1, IL-18, and IL-1β all regulate PCSK9 secretion by macrophages as well as in a host of tissues, including the liver, small intestine and kidney; on the other hand, MAPKs play a key role in regulating IL-1β-mediated PCSK9 secretion, particularly in the context of high-fat diet consumption (16). The study pointed out that macrophages secrete large amounts of PCSK9 *via* NLRP3 inflammasome activation. PCSK9 expression is downstream of NLRP3 and IL-1β, which are the major regulators of PCSK9 secretion. Furthermore, HFD-C results in the robust secretion of PCSK9, and this PCSK9 secretion is dependent on IL-1β upregulation, providing a strong link between IL-1β and PCSK9 in the proatherosclerotic and proinflammatory milieu (97, 98).

### Recent advances in the use of PCSK9 and NLRP3 inflammasome signaling as therapeutic targets for atherosclerosis

Inhibiting PCSK9 with inhibitors has been studied as a potential therapeutic approach in the field of atherosclerotic treatment (99). Different forms of PCSK9 inhibitors and their targets, including monoclonal antibodies (mAbs), small interfering RNAs (siRNAs), and vaccines, have recently been explored (100–102). At present, two mAbs against human PCSK9, alirocumab and evolocumab, are approved for listing and are available for use in clinical treatment (103). Ongoing clinical trials have shown that these PCSK9 mAbs are well tolerated, enhance the clearance of LDL-C, and decrease cardiovascular events in patients (104).Therapeutic monoclonal antibodies target PCSK9 in the plasma, while siRNA selective silences the translation of messenger RNA (mRNA) molecules, thus preventing the intracellular translation of PCSK9 mRNA to protein (105). Inclisiran is an siRNA and was discovered several years ago (106). The positive results of the ORION series provide strong supporting evidence for the clinical application of inclisiran in the treatment of atherosclerosis (107, 108). Compared with therapeutic mAbs, siRNA treatment shows a more sustained efficacy and requires fewer injections, thus overcoming some known barriers to treatment compliance compared with mAbs (109). The basic characteristic of a PCSK9 vaccine is the capacity to trigger the generation of anti-PCSK9 antibodies by the host (110). However, research on PCSK9 vaccines, such as PCSK9Qβ-003, AT04A and L-IFPTA+, is still in the animal experimental stage (44, 111, 112).

In terms of NLRP3 inhibitors, currently available clinical agents are targeting IL-1β (113). Although targeting IL-1β is an effective method for treating inflammatory diseases, it seems to have limitations. First, the activated NLRP3 inflammasome produces various inflammatory cytokines, including IL-1β, IL-18 secretion. All of cytokines play crucial roles in the occurrence and development of related diseases (114, 115). Second, IL-1β is produced not only by the NLRP3 inflammasome but also by other inflammasomes or in an inflammasome-independent manner, so inhibition of NLRP3 may have more immunosuppressive effects than inhibition of IL-1β (116, 117). Additionally, MCC950 can specific inhibit classical and nonclassical NLRP3 inflammasome activation and pro-inflammatory cytokines secretion to significantly decrease the maximum degree of aortic stenosis as well as the mean plaque size and volume and increase plaque stability (118–120). Several studies have revealed that MCC950 can directly interact with the Walker B motif in the NACHT domain to block ATP hydrolysis and inhibit NLRP3 inflammasome activation and formation (121, 122). In addition, CY-09, which is an analog of C172, was also found to directly bind to the ATP-binding site of the NLRP3 NACHT domain and inhibit its ATPase activity, inhibiting NLRP3 inflammasome oligomerization and activation (123). Besides, Song et al. revealed that blocking NLRP3 phosphorylation at S194 significantly decreased NLRP3 inflammasome activation, suggesting that the inhibition of NLRP3 phosphorylation may be a potential approach for treating NLRP3-related diseases (124). Melatonin has proven to be effective in treating atherosclerosis by inhibiting NLRP3 inflammasome signaling pathway (125, 126).

Collectively, the research on novel methods for inhibiting PCSK9 and NLRP3 inflammasome is still in an early stage, and the efficacy, safety and feasibility of these approaches require further study.

## Conclusions and perspective

In summary, PCSK9 can promote the occurrence and development of atherosclerosis by inhibiting the metabolism of LDL-C, enhancing the formation of foam cells and thus promoting the progression of vascular wall inflammation, suggesting that PCSK9 is an important intervention target in the occurrence and development of atherosclerosis. NLRP3 inflammasome activation contributes to the vascular inflammatory response driving atherosclerosis development and progression. Significantly, the effect of PCSK9 and the NLRP3 inflammasome on atherosclerosis seems to be synergistic. Interactions between PCSK9 and the NLRP3 inflammasome may form a positive feedback loop, acting together to drive the inflammatory response and lipid accumulation and thus promoting atherosclerosis. On the one hand, the NLRP3 inflammasome signaling pathway has been shown to promote PCSK9 secretion by regulating IL-1β, and this may be mediated by MAPK signaling. On the other hand, PCSK9 directly induces inflammatory responses in VSMCs as well as the expression and secretion of inflammatory factors and cytokines, including NLRP3 and its downstream molecules ASC, Caspase-1, IL-6, IL-18, and IL-1β, in macrophages. Collectively, these findings suggest a bidirectional positive correlation between the NLRP3 inflammasome signaling pathway and PCSK9. Therapeutic options that target PCSK9 and NLRP3 inflammasome may play an important role in the future treatment of atherosclerosis-related diseases. For example, inclisiran not only decreases LDL-C levels but also reduces Lp(a) concentrations. Given that Lp(a) is an independent risk factor for atherosclerosis, early treatment with inclisiran might result in extensive benefits for patients and decrease the damage caused by atherosclerosis *via* multiple mechanisms. Additionally, the inhibitors of NLRP3 inflammasome signaling that are currently available are agents that target IL-1β and caspase-1 as well as antagonists of the receptor P2X7.

It is noteworthy that knockout of the PCSK9 gene without inflammatory activation does not affect the levels of inflammatory proteins under physiological conditions; only the presence of factors that induce inflammatory responses, such as a high-fat diet, significantly decrease the levels of inflammatory cytokines. Besides, although the deficiency of the PCSK9 gene partially decreased inflammation in mice with atherosclerosis, it failed to diminish the inflammatory response completely. First, activation of the NLRP3 inflammasome during apoptosis is closely associated with mtROS-mediated mtDNA damage. Second, activated NLRP3-mediated IL-1β secretion in turn serves as a proinflammatory stimulant that can promote inflammatory responses. In addition, Chen et al. indicated that NLRP3, NLRP12 and NLRC4 exerted a significant synergistic effect, suggesting that NLRP3, NLRP12 and NLRC4 could be mutually regulated (127). This synergistic effect can promote caspase-1-dependent GSDMD cleavage-induced pyroptosis during vascular damage and accelerate the secretion of IL-1β. Finally, the synthesis of inflammatory factors and cytokines, such as ASC, Caspase-1, and IL-1β, may not be fully dependent on the NLRP3 inflammasome. NLRP6, NLRP7, NLRP12, NLRC4 and AIM2 have been shown to contribute to proinflammatory cytokine expression. As shown in the study by Liu et al., overexpression of NLRP6 enhanced the activation of caspase-1 and gasdermin-D, induced the pyroptosis of human gingival fibroblasts, and promoted the release of the proinflammatory mediator IL-1β (128).

In conclusion, the NLRP3 inflammasome signaling pathway and PCSK9 play critical roles in atherosclerosis and show promise as essential targets for the prevention and treatment of atherosclerosis. However, the precise mechanisms by which interactions between the NLRP3 inflammasome and PCSK9 affect atherosclerosis have largely remained unclear. Further research is needed to reveal the functions of PCSK9 and NLRP3 inflammasome signaling in atherosclerosis by revealing the underlying molecular mechanisms. Moreover, long-term clinical follow-up with a large sample size will still be necessary for studying PCSK9 and NLRP3 inhibitors since these inhibitors may increase the risk of diabetes and neurological diseases.

## Author contributions

YW and DF conceived the idea, analysis of literature, and writing of the manuscript. QY, JY, LW, and JW collected and read the literature and revised the article. MZ and ML read through and corrected the manuscript. All authors contributed to the article and approved the submitted version.

## Glossary

**Table:**

PCSK9	proprotein convertase subtilisin/kexin type 9
ER	endoplasmic reticulum
LDLR	low-density lipoprotein receptor
LDL-C	low-density lipoprotein cholesterol
MФ	macrophage
ox-LDL	oxidative low-density lipoprotein
LRP1	LDL-R-related protein 1
TLR4	toll-like receptors 4
NF-κB	nuclear factor kappa-B
IκBα	inhibitor kappa B alpha
ApoER2	apolipoprotein E receptor-2
ApoB48	apolipoprotein B-48
ACAT-2	acyl-coA cholesteryl acyl transferases 2
VSMCs	vascular smooth muscle cells
Bax	BCL2-associated X
Bcl-2	B-cell lymphoma-2
MAPK	mitogen-activated protein kinases
mTOR	mammalian target of rapamycin
VCAM-1	vascular cell adhesion molecule-1
ICAM-1	intercellular adhesion molecule-1
EC	endothelial cells
AP-1	activator protein-1
PRR	pattern recognition receptor
NLRP3	NOD-like receptor thermal protein domain associated protein 3
PAMPs	pathogen-associated molecular pattern
DAMPs	damage associated molecular patterns
LRR	leucine-rich repeat domain
NACHT	nucleotide triphosphatase domain
PYD	pyrin domain
CARD	caspase recruitment domain
TRIF	TRI domain-containing adaptor inducing interferon.B
MyD88	myeloid differentiation factor 88
IL-1β	interleukin-1β
IL-18	interleukin-18
GSDMD	gasdermin-D
GSDMD-NT	N-terminal cleaving product
Olfr2	olfactory receptor 2
mtROS	mitochondrial reactive oxygen species
HUVECs	human umbilical vein endothelial cells
P2X (4) R	purinergic receptor P2X4
P2X (7) R	purinergic receptor P2X7
IL-1Ra	interleukin-1 receptor antagonist
CagA	cytotoxin-associated protein
Oct-4	POU class 5 homeobox 1
KLF4	kruppel-like factor 4
ATP	adenosine-triphosphate
LPS	lipopolysaccharide
mtDNA	mitochondrial deoxyribonucleic acid
MAECs	mouse aortic endothelial cells
BMDCs	bone marrow-derived dendritic cells
HAoSMCs	human aortic smooth muscle cells
BMDMs	primary bone marrow-derived macrophages
HEK293T	human embryonic kidney 293T
ACS	acute coronary syndrome
PBMC	peripheral blood mononuclear cells
GLP-1R	glucagon like peptide 1 receptor
PKC	protein kinase C
NOX-4	NADPH oxidase 4
LDH	lactate dehydrogenase
CCL-2	chemokine ligand 2
CC	cholesterol crystals
UQCRC1	ubiquinol-cytochrome *c* reductase core protein 1
MI	myocardial infarct
HeFH	heterozygous familial hypercholesterolemia
SREBP-2	sterol element binding protein 2
CAT	a catalytic domain
CHD	coronary heart disease
ASCVD	atherosclerotic cardiovascular disease
IgG1	immunoglobulin G1
WAT	White Adipose Tissue
CVD	cardiovascular disease
NAFLD	non-alcoholic fatty liver diseases
NASH	non-alcoholic steatohepatitis
CD36	cluster of differentiation 36
LOX-1	lectin-like ox-LDL receptor 1
TNF-α	tumor necrosis factor-α
T2D	Type 2 diabetes
MI	myocardial infarction.
