# Supplementary figures and images for: Interactions between PCSK9 and NLRP3 inflammasome signaling in atherosclerosis
Source: Front Immunol. 2023 Feb 22;14:1126823. doi: 10.3389/fimmu.2023.1126823 (PMC9992811; doi:10.3389/fimmu.2023.1126823)

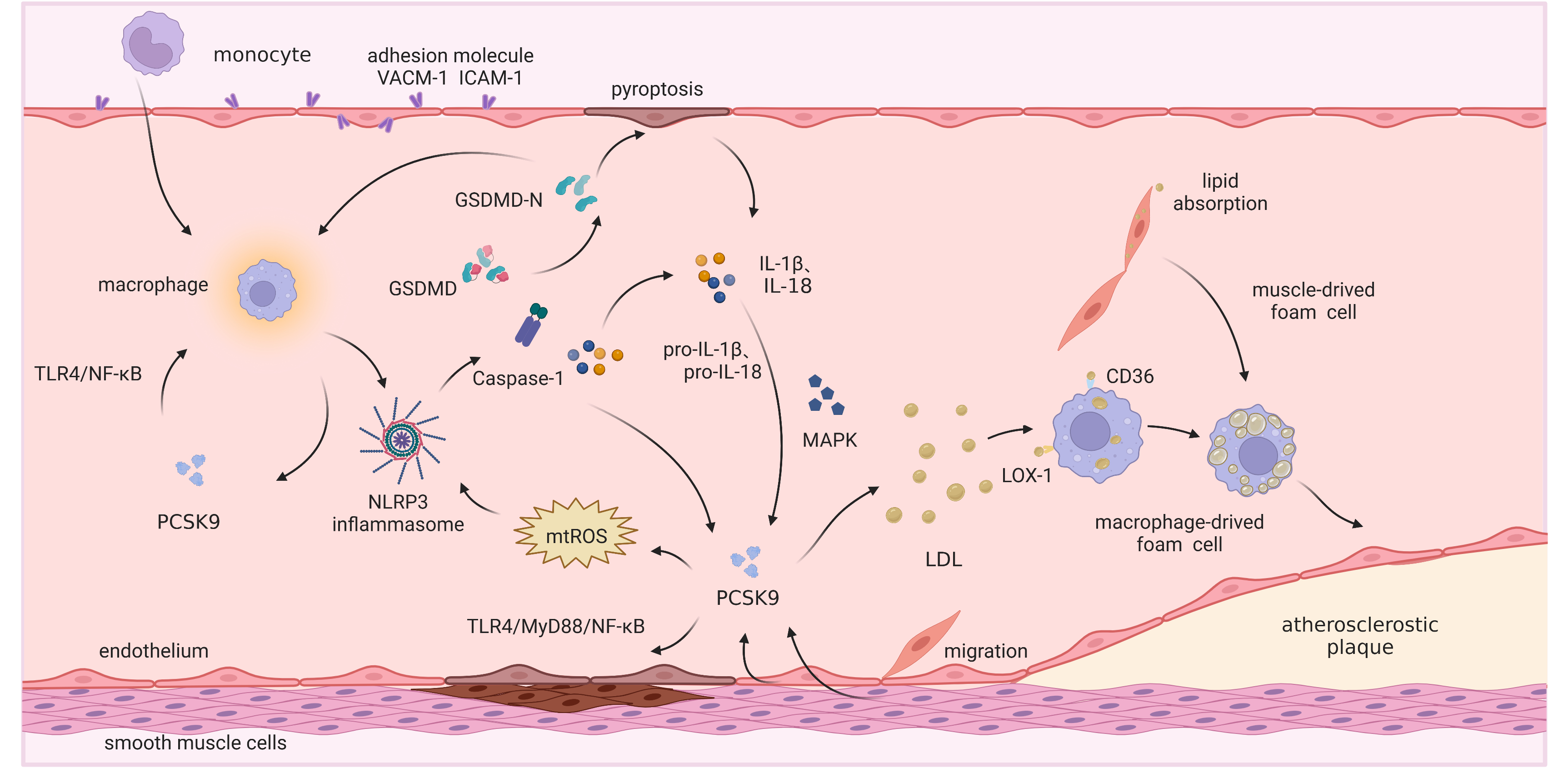

Supplement: Supplementary file 1 [file Image_1.jpeg]
